# Efficacy of Non-Invasive Brain Stimulation in Improving Working Memory in Children and Adolescents with Attention-Deficit/Hyperactivity Disorder: A Systematic Review

**DOI:** 10.3390/brainsci16050480

**Published:** 2026-04-29

**Authors:** Wilson Alexander Zambrano Vélez, Johanna Lilibeth Alcívar Ponce, Walter Gonzalo Bailón Bailón, Harol Marcial Castillo del Valle, Rocisela Adriana Baquerizo Quirumbay

**Affiliations:** Facultad de Ciencias Sociales y de la Salud, Universidad Estatal Península de Santa Elena, La Libertad 240250, Ecuador; jalcivar@upse.edu.ec (J.L.A.P.); wbailon1225@upse.edu.ec (W.G.B.B.); hcastillo@upse.edu.ec (H.M.C.d.V.); rossicelabaquerizo3@gmail.com (R.A.B.Q.)

**Keywords:** Attention-Deficit/Hyperactivity Disorder, non-invasive brain stimulation, transcranial magnetic stimulation, transcranial direct current stimulation, working memory, systematic review

## Abstract

**Highlights:**

**What are the main findings?**

- High-frequency rTMS (10 Hz) and anodal tDCS over the left dorsolateral prefrontal cortex (l-DLPFC) significantly improve executive working memory in pediatric ADHD.
- Clinical efficacy is more consistent in central executive components (N-back and backward digit span) than in simple phonological or visuospatial storage tasks.

**What are the implications of the main findings?**

- NIBS acts as a “neuroplasticity primer” that can partially correct functional recruitment deficits in the frontoparietal network of children and adolescents.
- Both tDCS and rTMS demonstrate a superior safety-to-efficacy ratio compared to pharmacological side effects, supporting their role as viable complementary pediatric therapies.

**Abstract:**

**Background/Objectives**: Attention-Deficit/Hyperactivity Disorder (ADHD) is associated with working memory deficits linked to frontoparietal alterations. Non-invasive brain stimulation (NIBS) is a potential intervention to modulate neuroplasticity and improve this executive function. In this study, we aimed to evaluate the clinical efficacy of non-invasive brain stimulation techniques (tDCS/rTMS) for strengthening working memory in children and adolescents with ADHD. **Methods**: This systematic review adhered to the PRISMA 2020 guidelines, with a search of Scopus and Web of Science conducted to identify relevant studies published between 2011 and 2026. Eligibility criteria, defined a priori, included original empirical studies (RCTs and quasi-experimental designs) focusing on pediatric populations (≤18 years) diagnosed with ADHD. Eligible interventions involved tDCS or rTMS with explicit working memory outcomes. Only peer-reviewed articles published in English or Spanish were included. Reviews, case reports, and studies exclusive to adults were excluded. Data on application parameters, durability, and safety were extracted for narrative synthesis. **Results**: Six studies met the criteria. Both tDCS and rTMS targeting the left dorsolateral prefrontal cortex showed improvements in working memory, particularly in executive components measured using digit span backward and N-back tasks. High-frequency rTMS (10 Hz) with repeated sessions showed more consistent effects, while tDCS showed modest and variable improvements. Evidence regarding long-term effects was limited. Both techniques were well-tolerated, with mild and transient adverse events. **Conclusions**: NIBS shows promise as a complementary intervention to improve working memory in pediatric ADHD; however, current evidence is limited. Larger, standardized, longitudinal trials are required to confirm its efficacy and clinical utility.

## 1. Introduction

Attention-Deficit/Hyperactivity Disorder (ADHD) is a neurodevelopmental disorder of multiple etiology with a solid genetic basis, non-hereditary factors, and interaction with environmental factors [1,2]. It presents a persistent pattern of inattention, hyperactivity, and impulsivity [3,4]. It is most frequently identified in the child and adolescent population [5,6], although its clinical manifestations are also typically diagnostic well into adulthood. Globally, the prevalence of ADHD is estimated at approximately 5% [7], reaching 7.2% in children and adolescents [8]. However, reported rates vary widely, ranging from 2% to 18%, largely due to heterogeneity in diagnostic criteria, methodological approaches, assessment instruments, and population characteristics across different studies [9].

Of particular note, ADHD has been associated with difficulties in key domains of executive functions (EFs) [10], including working memory (WM) [11,12,13,14], which is considered a cognitive marker [15] that distinguishes individuals with the disorder from non-affected individuals [16]. WM refers to a neurocognitive system responsible for the temporary storage and active manipulation of information necessary for complex cognitive processes such as reasoning, comprehension, and goal-directed behavior [6,17]. The most influential framework for understanding WM is Baddeley’s multicomponent model [18,19], which has provided a solid theoretical and methodological foundation for investigating WM in neurodevelopmental disorders, including ADHD [20].

According to this model, WM consists of three main components: a general-domain central executive and two specific-domain subsystems: phonological short-term memory and visuospatial short-term memory [21,22]. The central executive governs attentional control, continuous updating, dual-task coordination, and the temporal sequencing of information, whereas the phonological subsystem preserves verbal and auditory–linguistic material, and the visuospatial subsystem retains non-verbal visual and spatial representations. These components are considered functionally dissociable and are supported by partially distinct neural substrates [22,23,24].

WM is fundamentally based on the active and sustained maintenance of information in the prefrontal cortex (PFC). This region does not operate in isolation but interacts dynamically with parietal areas depending on task demands, forming a frontoparietal network that facilitates temporary storage and information manipulation according to contextual requirements [25]. Neuroimaging evidence indicates that individuals with ADHD exhibit reduced neural activation in this network, especially in the prefrontal and parietal cortices, during tasks requiring working memory [26]. This attenuated recruitment of frontoparietal regions suggests functional inefficiency in the neural systems underlying working memory processes in this population.

The results of previous studies have shown that alterations in this system in ADHD are closely related to poor academic performance and a reduced capacity to complete complex tasks [27,28,29], findings widely replicated in children, adolescents, and young adults [30]. These deficits are also related to inattention [31], deficient inhibition, and mental organization [32] and extend to problems in daily functioning and poorer long-term outcomes, signaling the need for specific interventions beyond standard pharmacological treatment [25,33]. Although medications are effective, side effects and long-term use have driven a growing interest in non-pharmacological and neuromodulatory approaches [34].

Non-invasive brain stimulation (NIBS) is increasingly used to promote neurological or psychiatric rehabilitation by modulating neural plasticity [35,36]; furthermore, it has emerged as a promising therapeutic approach for ADHD [37,38]. Research primarily highlights repetitive transcranial magnetic stimulation (rTMS) [39,40] and transcranial direct current stimulation (tDCS) [41,42,43]. These methods are promising because they can stimulate key brain dysfunctions established in ADHD during the last two decades of functional magnetic resonance imaging (fMRI) research [37,44,45]. NIBS has emerged as a promising neuromodulatory approach to enhance neuroplasticity within the frontostriatal and frontoparietal circuits involved in ADHD [46].

However, despite the rapid expansion of the literature, several critical research gaps remain. Existing evidence demonstrates no widespread consensus regarding NIBS that collectively identifies the application protocols for tDCS and TMS to maximize WM improvement in pediatric and adolescent ADHD populations. Gaps also exist regarding the durability of NIBS-induced WM gains—including medium- or long-term follow-up—and the comprehensive assessment of safety and adverse events. To address these limitations, this study represents, to the authors’ knowledge, the first systematic review in which the clinical efficacy of both tDCS and rTMS is concurrently integrated and compared, specifically focused on working memory within the pediatric and adolescent ADHD population. While the authors of previous studies often address these techniques in isolation or within adult cohorts, herein, we provide a novel, comparative analysis of mechanistic principles and protocol-specific outcomes tailored to the neurodevelopmental window of minors.

In this systematic review, we therefore posed the following Research Question (PICO): To what extent does the use of non-invasive brain stimulation (I), compared to pharmacological treatment or placebo (C), improve working memory capacity (O) in pediatric and adolescent populations diagnosed with ADHD (P)? In this context, the aim was to evaluate the clinical efficacy of non-invasive brain stimulation techniques (tDCS/rTMS) for strengthening working memory in children and adolescents with ADHD. To complement this development, specific objectives were established: to identify the application protocols (cortical areas and frequency) that report higher rates of therapeutic success; to analyze the durability of post-intervention cognitive effects according to the reported evidence; and to determine the safety and adverse effects recorded in the use of these technologies in minors. By focusing on working memory as a central cognitive outcome within a PICO-based framework, we seek to inform the rational design of neuromodulatory interventions and clarify the clinical potential and current limitations of non-invasive brain stimulation as a complement or alternative to medication in pediatric ADHD.

## 2. Materials and Methods

### 2.1. Study Design

This study is a systematic review aimed at evaluating the efficacy of non-invasive brain stimulation (NIBS), specifically transcranial direct current stimulation (tDCS) and repetitive transcranial magnetic stimulation (rTMS), in improving working memory in pediatric and adolescent populations diagnosed with ADHD.

The review was designed and is presented in accordance with the PRISMA 2020 (Preferred Reporting Items for Systematic Reviews and Meta-Analyses) guidelines proposed by Page et al. [47] to ensure transparency, reproducibility, and methodological rigor. Additionally, a PRISMA flow diagram was developed to systematically document the identification, screening, and inclusion of studies.

The protocol for this systematic review was registered in the Open Science Framework (OSF) with the identifier https://doi.org/10.17605/OSF.IO/G8ZTP.

### 2.2. Unit of Analysis

The unit of analysis consisted of original empirical studies published between January 2011 and February 2026. The selection of this timeframe is justified by the need to capture the period of greatest innovation and clinical consensus in the use of NIBS techniques in the field. This interval is characterized by the refinement of neuroimaging-guided targeting and the establishment of international safety guidelines specifically for the developing brain. This span integrates the surge in research following the publication of standardized safety protocols for pediatric and youth populations and coincides with the current updated diagnostic criteria of the DSM-5, ensuring that the findings possess maximum relevance and applicability in the contemporary clinical context. Under this currentness criterion, the effects of tDCS or rTMS on working memory performance were evaluated in pediatric or adolescent populations (≤18 years) diagnosed with ADHD according to DSM or ICD criteria. Only original articles, randomized controlled trials (RCTs), controlled clinical trials, and quasi-experimental studies were considered. The final sample comprised studies retrieved from the Scopus and Web of Science (WoS) databases that met all predefined inclusion criteria.

### 2.3. Procedure

The review process was organized into four structured phases, following a systematic and replicable protocol.

#### 2.3.1. Phase I: Planning

During this phase, the research problem was conceptually defined and delimited through the PICO framework: Population (P), Children and adolescents (≤18 years) diagnosed with ADHD; Intervention (I), non-invasive brain stimulation techniques (tDCS or rTMS), with clearly reported stimulation parameters (cortical target area, intensity, frequency for rTMS, current intensity for tDCS, session duration, and number of sessions); Comparator (C), Pharmacological treatment, sham stimulation, placebo, or passive control group; Outcome (O), Primary outcome: changes in working memory performance measured using validated neuropsychological instruments. Secondary outcomes included protocol characteristics associated with greater efficacy, durability of effects (short, medium, or long-term follow-up), and reported adverse events.

Inclusion and exclusion criteria were defined a priori to reduce selection bias.

Inclusion Criteria:

- Original empirical studies (RCTs, controlled designs, or quasi-experimental).
- Pediatric/adolescent population with ADHD.
- Interventions using tDCS or rTMS.
- Explicit evaluation of working memory as an outcome.
- Peer-reviewed publications indexed in Scopus or WoS.
- Articles published in English or Spanish between 2011 and 2026.

Exclusion Criteria:

- Reviews, meta-analyses, case reports, editorials, or letters.
- Studies conducted exclusively in adults (>18 years).
- Studies that do not report specific working memory results.
- Publications in languages other than English or Spanish.

#### 2.3.2. Phase II: Data Collection

The literature search was conducted on 16 February 2026, exclusively targeting the Scopus and Web of Science (WoS) databases. This deliberate focus is justified by the Gold Standard status of these platforms, which represent the most rigorous filters for scientific quality globally. Unlike other repositories, Scopus and WoS apply strict inclusion criteria based on citation impact and peer-review transparency, ensuring that the synthesized evidence originates from journals with high editorial standards. Methodologically, it is noted that Scopus and WoS provide a comprehensive overlap (estimated at over 90%) with MEDLINE and other specialized databases in the fields of neuroscience and child psychology; therefore, their use as primary sources ensures the retrieval of all high-impact clinical trials while minimizing the inclusion of low-evidence publications.

Furthermore, we intentionally excluded gray literature (such as doctoral theses, conference abstracts, or non-indexed reports) to prioritize “Methodological Stability.” In the context of pediatric ADHD and neuromodulation, where protocol precision (intensity, frequency, and safety) is critical, gray literature often lacks the rigorous peer-review validation necessary to inform clinical practice reliably. By restricting the search to the highest-tier indexed journals, we establish a baseline of “Verified Evidence,” ensuring that the protocol recommendations for tDCS and rTMS are based on data that have undergone strict independent scrutiny, thus reducing the risk of reporting bias inherent in non-standardized publications.

A structured search strategy was developed using controlled vocabulary and free-text terms related to the topic. Additionally, Boolean operators (AND/OR) and parentheses were applied to ensure logical precision and avoid ambiguity in operator precedence. Subsequently, filters were applied to restrict results to peer-reviewed journal articles published in English or Spanish within the last 15 years. All retrieved records were exported to a Mendeley Reference Management (v.2.110.0; Mendeley Ltd., London, UK) file for subsequent processing. The detailed search strategy can be found in Supplementary Material File S1.

#### 2.3.3. Phase III: Organization, Selection, and Analysis

**Figure 1:**
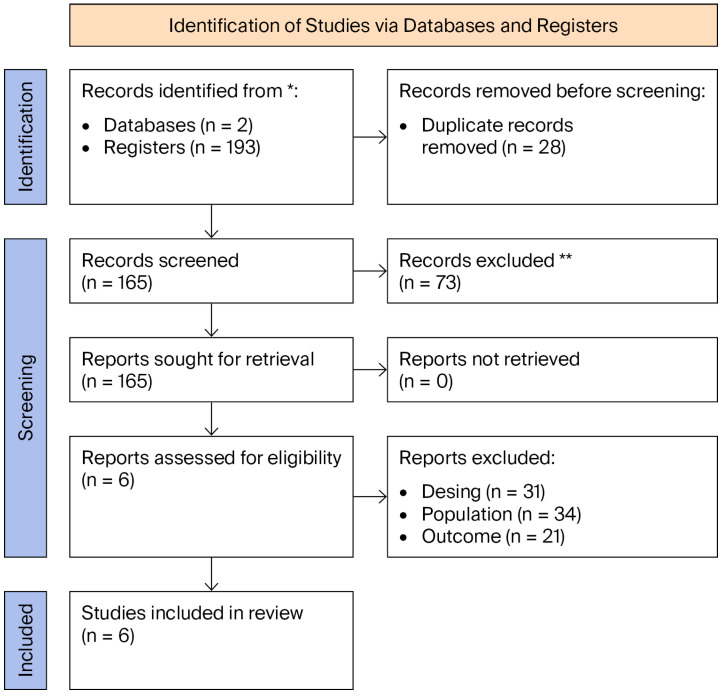
PRISMA Flow Diagram. Note: * Records identified from databases include Scopus and Web of Science. ** Records excluded by human reviewers during the screening phase (title/abstract).

The study selection process followed the PRISMA model and was carried out in three sequential steps, documented in the flow diagram (Figure 1):

- Identification: A total of 193 records were compiled from the examined databases (*n* = 2), Scopus and WoS. Before screening, 28 duplicate records were removed, leaving 165 unique records for the initial evaluation phase.
- Screening: Two independent reviewers screened 165 titles and abstracts, with 73 irrelevant records excluded at this stage. No studies were reported as not retrieved. Subsequently, during the eligibility evaluation (full-text), 86 articles were excluded based on predefined inclusion and exclusion criteria for the following reasons: irrelevant methodological design (*n* = 31), mismatched population (*n* = 34), and outcomes not aligned with the study objective (*n* = 21).
- Inclusion: Six studies that met all methodological criteria were selected. Despite the small sample size, these studies represent the highest level of available evidence for this specific neurocognitive outcome.

To organize the data, a standardized extraction matrix was created in Excel (v.16.0; Microsoft Corp., Redmond, WA, USA). The following variables were extracted: Author and year of publication, country of study, methodological design, sample size and age range, type of NIBS (tDCS or rTMS), stimulated cortical area, stimulation parameters (intensity, frequency, duration, and number of sessions), working memory evaluation instruments, main results, post-intervention follow-up, and reported adverse events.

Additionally, methodological quality and risk of bias were independently assessed by two reviewers using the Cochrane Risk of Bias tool for randomized trials (RoB 2) and the Risk of Bias in Non-randomized Studies of Interventions (ROBINS-I) for quasi-experimental designs. Five domains were evaluated: randomization process, deviations from intended interventions, missing outcome data, measurement of the outcome, and selection of the reported result. Discrepancies were resolved through consensus with a third senior author.

Given the anticipated heterogeneity, the findings were synthesized narratively through a structured analysis, grouping studies by stimulation modality (tDCS vs. rTMS) regarding application protocols reporting higher therapeutic success rates, durability of post-intervention cognitive effects, and the safety and adverse effects recorded in minors.

#### 2.3.4. Phase IV: Presentation of Results

In the final phase, results were organized and presented according to the following:

- Magnitude and direction of improvements in working memory (efficacy).
- Application protocols (cortical areas and frequency) reporting higher therapeutic success rates.
- Durability of post-intervention results.
- Safety profile and reported adverse effects.

### 2.4. Methodological Rigor and Reproducibility

All methodological decisions were predefined to minimize selection and reporting bias. The structured PICO framework, explicit eligibility criteria, independent selection, standardized data extraction, and adherence to the PRISMA 2020 guidelines reinforce the internal validity and reproducibility of this systematic review.

### 2.5. Declaration of Generative AI and AI-Assisted Technologies

During the preparation of this work, the authors used the Scopus AI (2026 Edition; Elsevier B.V., Amsterdam, The Netherlands) tool to assist in the identification, search, and expansion of relevant literature within the field of non-invasive brain stimulation and ADHD. This technology was employed to optimize the literature retrieval process and ensure a comprehensive coverage of the contemporary clinical consensus. Additionally, the Gemini 1.5 Pro language model (v.1.5; Google LLC, Mountain View, CA, USA) was utilized for the translation of the original manuscript from Spanish into English.

Following the use of these tools, the authors methodically reviewed, edited, and validated the search results and the technical accuracy of the translated text to ensure it met academic standards. The authors take full responsibility for the integrity and final content of the published work.

## 3. Results

### 3.1. Synthesis of Included Studies

Due to the observed heterogeneity in technical parameters, assessment instruments, and follow-up duration, findings were synthesized through a structured narrative analysis (Table 1). Studies were grouped by stimulation modality: Transcranial direct current stimulation (tDCS) and repetitive transcranial magnetic stimulation (rTMS).

### 3.2. Risk of Bias in Included Studies

The systematic assessment of the risk of bias (Table 2) reveals that 66.7% of the included studies (*n* = 4) present a “Low Risk” of bias across most domains. However, 33.3% (*n* = 2) were classified as having “Some Concerns” or “High Risk” in specific areas, primarily related to attrition and missing outcome data. Through our analysis, we found that the study by Wang et al. [49] presented a higher risk due to a 12.5% loss of participants during follow-up without a clear intention-to-treat analysis. In the study by Guimarães et al. [53], some concerns were noted regarding the pre-registration of the protocol. Despite these individual limitations, the collective evidence maintains a methodological stability that allows for a reliable narrative synthesis, although the lack of long-term blinding remains a transversal limitation in the field.

### 3.3. Comparative Efficacy and Mechanistic Analysis

Beyond a simple listing, the synthesis of outcomes indicates specific patterns of response based on the stimulation modality:

- rTMS and Executive Control: High-frequency (10 Hz) rTMS protocols [48,50] reported improvements primarily in the central executive component (backward digit span) over the Phonological Loop (forward span). In the study utilizing fNIRS [49], behavioral gains in N-back tasks coincided with an increase in oxyhemoglobin (HbO) levels, specifically in the L-DLPFC.
- tDCS Efficacy and Parameters: tDCS showed positive effects in acute, single-session settings [52]. However, no significant differences were found in multi-session protocols [53], even when using a higher intensity of 2 mA.

### 3.4. Analysis of Protocol Parameters and Therapeutic Success

The data identify the L-DLPFC as the most frequent target area. Outcome success was documented in the following conditions:

- Success Factors: Protocols utilizing 10–15 sessions of 10 Hz rTMS at 80–100% motor threshold [48,50] yielded the most consistent significant improvements (*p* < 0.05) within the sample.
- Domain Specificity: The absence of significant effects in visuospatial components (Corsi Block-Tapping) was reported in studies targeting the L-DLPFC [53].

### 3.5. Durability and Neuroplastic Stability

A critical gap in the descriptive-to-analytical transition is the temporal stability of effects. Current evidence [48,51,53] is predominantly acute-phase focused. Analytically, the absence of longitudinal follow-up prevents the confirmation of whether these interventions induce true Long-Term Potentiation (LTP) or merely a transient state of altered cortical excitability. While rTMS at 10 Hz is theoretically capable of driving synaptic strengthening through repetitive depolarization, the studies reviewed primarily report outcomes measured within 24 to 48 h post-intervention, leaving the “wash-out” period uncharacterized in this pediatric population.

Furthermore, the transition from temporary neuromodulation to stable neuroplastic reorganization remains unproven in the ADHD context. In the absence of mid-term data (3–6 months) to assess the persistence of executive gains, NIBS cannot yet be classified as a disease-modifying treatment. Current results suggest it operates as a temporary cognitive enhancer that may require periodic “booster” sessions to maintain the functional state of the frontoparietal networks, as the developing brain’s homeostatic plasticity may counteract short-term exogenous stimulation.

### 3.6. Reported Adverse Events and Safety Results

From an analytical standpoint, the safety data [48,51,52] confirm that the pediatric plasticity window can be safely navigated using adult-derived safety margins. The events reported (mild headache, pruritus, and transient erythema) were localized and non-cumulative, suggesting that NIBS possesses a superior safety-to-efficacy ratio compared to certain pharmacological side effects, supporting its role as a viable complementary therapy.

## 4. Discussion

### 4.1. Efficacy of NIBS on Working Memory Components

The findings suggest that both rTMS and tDCS applied over the left DLPFC may potentially produce improvements primarily in the central-executive component of working memory, as evidenced by tasks such as the backward digit span and N-back. In contrast, effects on phonological or visuospatial storage were inconsistent. This trend emphasizes a facilitatory modulation of prefrontal excitability capable of optimizing updating processes and attentional control. However, these observations are based on a restricted number of studies (*n* = 6), which precludes definitive generalizations.

Mechanistically, it has been proposed that rTMS achieves this effect through electromagnetic induction that triggers Long-Term Potentiation (LTP)-like plasticity, effectively increasing excitatory neurotransmission in the hypoactive prefrontal circuits of the ADHD brain (Hallett, 2007; Hoogman et al., 2019) [54,55]. In parallel, tDCS operates by shifting neuronal membrane potentials toward depolarization through anodal stimulation, which lowers the threshold for neuronal firing and facilitates “online” cognitive recruitment during executive tasks (Stagg & Nitsche, 2011) [56]. Nevertheless, the current evidence from the included studies provides only an indirect suggestion of these processes rather than a direct confirmation of LTP-like plasticity in this specific population.

This finding stands in contrast to studies in which the authors conclude that excitatory stimulation of the DLPFC, especially through high-frequency rTMS, preferentially enhances the executive components of working memory measured with N-back tasks, whereas effects on other cognitive domains are less consistent [57,58]. In the meta-analysis by Brunoni & Vanderhasselt [57], rTMS over the DLPFC was associated with improvements in both accuracy and reaction times in N-back, whereas tDCS showed more modest effects centered on response speed without a clear gain in precision, which converges with the greater robustness observed in the 10 Hz protocols of this study. Similarly, Bagherzadeh et al. [58] demonstrated that high-frequency rTMS over the left DLPFC improves performance in verbal digit span and a 2-back visuospatial task, but not in a simple visuospatial span, supporting the notion of a facilitatory modulation of updating and attentional control processes rather than mere phonological or visuospatial storage. Nevertheless, as described in both works, the heterogeneity in stimulation parameters, design, and sample sizes limits the possibility of establishing firm conclusions on conclusive superiority between techniques or on the generalization of effects to all subcomponents of working memory.

### 4.2. Protocol Standardization and Neurobiological Plausibility

Regarding the most effective application protocols, the convergence on the stimulation of the left DLPFC reinforces the neurobiological plausibility of the approach, given its role in the frontoparietal working memory network. In rTMS, the most consistent protocols included high frequency (10 Hz), intensities between 80 and 100% of the motor threshold, and schedules of 10–15 sessions, suggesting a possible cumulative effect. In tDCS, anodal montages over F3 with 1–2 mA showed favorable results when applied in multiple sessions; however, the absence of effects in some studies with similar parameters indicates that the response may depend on individual variables and the clinical context, underlining the need for standardization and personalization.

This is situated against previous works in pediatric populations with ADHD that convergently show that prefrontal stimulation requires careful definition of parameters and that its effects are sensitive to both localization (DLPFC vs. other frontal regions) and the number of sessions and individual characteristics. The results of a systematic review and meta-analysis by [44,45] on rTMS and tDCS in ADHD (children and adults) highlight that they stimulate the DLPFC (mainly left) but that protocols usually include only 1–5 sessions and show small or trend-level clinical and cognitive effects, with high variability linked to frequency, intensity, and number of sessions. This evidence specifically suggests that multi-session protocols better adjusted to the individual could produce greater cumulative effects, although current evidence does not yet allow for the definition of an optimal standard [45].

Beyond technical parameters, the pharmacological status of participants emerges as a significant source of clinical heterogeneity. Psychostimulants, standard in ADHD treatment, modulate dopamine and norepinephrine levels—catecholamines that play a documented role in gating the cortical plasticity induced by NIBS. The inclusion of studies in which medication was suspended [48,51,53] alongside those with stable medication use [49,52] introduces a potential confounding variable. Concomitant medication may either ‘ceiling’ the effects of stimulation or, conversely, act synergistically. This interaction remains an underexplored moderator that complicates the direct attribution of executive gains solely to the stimulation protocols.

Taken together, these findings provide evidence that the choice of the left DLPFC as a target is neurobiologically plausible but does not guarantee beneficial effects if the montage and parameters are not optimal; furthermore, effects appear to depend on multi-session protocols that allow for a possible cumulative effect. The notable inter-individual variability and differences between studies make it essential to move toward greater methodological standardization and designs that contemplate dose personalization and the clinical context.

### 4.3. Durability of Effects and Neuroplastic Stability

Corresponding to the durability of the effects, evidence regarding the persistence of improvements is limited, as the authors of most studies evaluated only the immediate post-intervention effect without structured medium- or long-term follow-up. This evidence is supported by studies conducted in pediatric populations with ADHD; in the randomized controlled trial by Westwood et al. [59], the authors report that, although immediate improvements in working memory tasks are observed following the application of these techniques, they are not consistently maintained in subsequent evaluations. This evidence indicates that the effects correspond to short-lived functional changes in cortical excitability rather than stable neuroplastic modifications. This limitation reduces the clinical extrapolation of the findings and constitutes one of the main gaps in the current literature.

### 4.4. Safety Profile and Pediatric Tolerability

Regarding the safety and adverse events of these techniques, study authors consistently report a favorable safety profile for both techniques in the pediatric population, with mild and transient adverse effects such as local pruritus or erythema in tDCS and mild headache in rTMS, with no records of serious events. These results support the tolerability of NIBS under standardized parameters; however, considering the neurodevelopmental stage of the participants, systematic monitoring and longitudinal studies evaluating cumulative safety are required.

Recent evidence expands upon the interpretation presented. The results of a systematic review and meta-analysis by Zhang et al. [60] indicate that these techniques in children and adolescents with ADHD present predominantly mild and transient adverse events, with no records of serious effects; moreover, in the randomized clinical trial by Schertz et al. [61], the authors report occasional headache and mild discomfort as the main side effects, with good overall tolerability. Likewise, the safety review of NIBS in pediatric populations by Salehinejad & Siniatchkin [62] confirms that both tDCS and rTMS are well-tolerated under standardized protocols, without severe adverse events. Together, the findings of these studies are consistent with the proposed findings and reinforce the need for longitudinal studies to evaluate cumulative safety in neurodevelopment.

### 4.5. Theoretical and Clinical Implications: Filling the Research Gap

The novelty of this systematic review lies in its integrated comparative approach, representing one of the first academic efforts to concurrently evaluate tDCS and rTMS protocols specifically for working memory in the pediatric ADHD population. Although the small sample size limits the strength of the conclusions, our findings reveal that while both share a neurobiological target (L-DLPFC), high-frequency rTMS offers more consistent preliminary evidence for strengthening executive control, whereas tDCS shows higher sensitivity to individual baseline states. These findings contribute to a conceptual framework that aims to explore how NIBS might address functional recruitment deficits in the frontoparietal network. Furthermore, we move beyond general ADHD symptoms to isolate working memory as a primary cognitive outcome, offering researchers and clinicians a roadmap for the rational design of multi-session interventions that acknowledge the unique neuroplastic window of children and adolescents.

### 4.6. Limitations and Future Directions

The present systematic review is constrained by limitations that must be considered when interpreting the findings. The number of included studies was small, and they comprised small sample sizes, limiting statistical power and the generalization of results. Furthermore, marked clinical and methodological heterogeneity exists among the studies, evidenced by differences in age, ADHD severity, pharmacological status, and the presence of comorbidities, in addition to differences in technical stimulation parameters and the instruments used to measure working memory. A substantial limitation is the lack of systematic control over the moderating role of concomitant medication. Considering that ADHD drugs alter the threshold of neuronal excitability and synaptic weight, the clinical heterogeneity between studies that permit continued medication use and those that do not represents a significant source of variance. This discrepancy hinders the ability to determine whether the observed neuroplasticity (LTP-like effects) is a pure result of NIBS or a product of an uncontrolled pharmacodynamic interaction, a factor that the current literature has not yet adequately addressed.

The authors of future studies should aim to strengthen the empirical evidence through more consolidated and standardized methodological designs. Therefore, given the size of the selected sample, it is evident that randomized clinical trials with large sample sizes are required to improve external validity and statistical power. It is essential to standardize stimulation protocols, precisely defining optimal parameters for frequency, intensity, duration, and number of sessions, in addition to reinforcement schemes to evaluate cumulative effects. Additionally, longitudinal medium- and long-term follow-ups should be incorporated to determine the persistence of cognitive effects and their impact on academic and daily functioning. It is a priority to explore combined approaches that integrate brain stimulation with cognitive or psychoeducational interventions, evaluating possible synergistic effects. Similarly, research must move toward the personalization of treatments using neurophysiological biomarkers and individual cognitive profiles of ADHD. It is also recommended to conduct direct comparisons between tDCS and rTMS and explore different cortical targets to determine the relative superiority of each technique. Furthermore, it is necessary to strengthen long-term safety evaluation in the pediatric population, combined with the adoption of open science practices that increase the transparency and reproducibility of studies.

## 5. Conclusions

The study of the efficacy of non-invasive brain stimulation (NIBS) on working memory in pediatric and adolescent populations with ADHD is a recent field of research that has shown progressive growth, despite the still limited number of available empirical studies. Within the context of this systematic review, only six investigations meeting the inclusion criteria were identified, demonstrating that scientific production remains incipient and highlighting a clear need to expand research in this area.

According to the analyzed results, both tDCS and rTMS show potential for a positive effect on performance in working memory tasks, particularly regarding the central-executive component evaluated through tasks such as backward digit span and N-back. Nevertheless, this evidence cannot be considered a definitive confirmation due to the magnitude of these effects varying between studies and depending on methodological factors. The small evidence base currently prevents the establishment of a robust mechanistic model or a confirmed clinical protocol.

Variables of clinical interest—such as ADHD subtype, the presence of comorbidities, pharmacological status, or differences in neurocognitive development—are not systematically reported or controlled in most of the included studies. The influence of these factors on the response to NIBS remains uncertain, despite the fact that evidence suggests they can significantly modulate cognitive performance and therapeutic efficacy, both in daily life and in specific neuropsychological tests of working memory.

The instruments used to evaluate working memory were mostly standardized neuropsychological tests focused on specific system components, such as phonological storage, executive control, or visuospatial processing. However, these measures prevent a comprehensive assessment of functioning in complex ecological contexts that demand the integration of multiple executive processes; therefore, the incorporation of ecological or functional assessments may provide a broader understanding of the real-world impact of these interventions on the daily lives of patients with ADHD.

Regarding safety, available evidence indicates that both techniques present a favorable tolerability profile in child and adolescent populations, with mild and transient adverse effects and no records of serious events. Nonetheless, given the ongoing neurobiological development within this population, it is essential to continue investigating long-term safety and the potential cumulative effects of NIBS through rigorous longitudinal designs that ensure its safe clinical applicability.

## Figures and Tables

**Table 1:** Systematization of Selected Studies.

Study	Technique and Design	Participants	Stimulation Parameters	WM Component and Assessment	Main Outcomes (WM)	Safety and Follow-Up
Qian et al. [48]	rTMS (10 Hz)/RCT, Double-blind	*n* = 48 (24 in the treatment group and 24 in the control group); 6–10 y; ADHD (DSM-5); Meds suspended	L-DLPFC; 80–100% MT; 20 min/sess; 10 sessions total	Central Executive: Digit Span (backward)	Significant improvement vs. sham (*p* < 0.05). Moderate effect size	Mild headache. Post-immediate evaluation only
Wang et al. [49]	rTMS (10 Hz)/ Experimental (fNIRS)	*n* = 40 (20 treatment and 20 control); however, 5 were lost during follow-up, resulting in a final selection of 35 cases (17 treatment, mean age 8.45 ± 1.53 y; 18 control, mean age 8.23 ± 1.42 y); ADHD; Stable meds	L-DLPFC; 90% MT; 20 min/sess; 10–15 sessions	Central Executive: N-back task	Significant HbO increase in DLPFC. Modest improvement in N-back accuracy (*p* < 0.05)	Good tolerability. No longitudinal follow-up
Nagy et al. [50]	rTMS (10 Hz)/RCT, Sham-controlled	*n* = 60; 6–12 y; ADHD (DSM-IV/5); Meds variable	L-DLPFC; 90% MT; 1000–2000 pulses; 10–15 sess	Central Executive: Digit Span (backward)	Significant improvement in backward span vs. sham (*p* < 0.05)	Mild transient headache. No mid-term persistence evidence
Krauel et al. [51]	tDCS/RCT	*n* = 60; 10–18 y; ADHD; Meds suspended	L-DLPFC (F3) Anode; 1 mA; 20 min; 5–10 sessions	Central Executive: N-back task	Significant but modest improvement in accuracy (*p* < 0.05)	Mild pruritus/erythema. Immediate post-test only
Nejati et al. [52]	tDCS/Crossover, Double-blind	*n* = 25; Adolescents; age M_1_ = 10, SD_1_ = 2.23; M_2_ = 9, SD_2_ = 1.8; DSM-IV; Stable meds	L-DLPFC (F3) Anode; 1 mA; 15 min; Single session	Attentional Control: N-back (1-back)	Significant accuracy increase and RT reduction (*p* < 0.05)	Mild tingling. Acute effect only (no maintenance)
Guimarães et al. [53]	tDCS/Crossover, Triple-blind	*n* = 18; 6–16 y; ADHD (DSM-5); Meds suspended	L-DLPFC (F3) Anode; 2 mA; 30 min; 5 sessions	Verbal, Executive, Visuospatial: Digit Span, Corsi	No significant differences vs. sham in any WM measure	Mild erythema. No significant immediate effect

**Table 2:** Risk of Bias Assessment Summary.

Study	Randomization/ Selection	Deviations from Intervention	Missing Outcome Data	Outcome Measurement	Reported Result	Overall Bias
Qian et al. [48]	Low	Low	Low	Low	Low	Low Risk
Wang et al. [49]	Some concerns	Low	High Risk	Low	Low	Some concerns
Nagy et al. [50]	Low	Low	Low	Low	Low	Low Risk
Krauel et al. [51]	Low	Low	Low	Low	Low	Low Risk
Nejati et al. [52]	Low	Low	Low	Low	Low	Low Risk
Guimarães et al. [53]	Low	Low	Low	Low	Some concerns	Some concerns

## Data Availability

The data supporting the findings of this systematic review, including the study protocol, search strategies, and the data extraction matrix, are openly available in the Open Science Framework (OSF) at https://doi.org/10.17605/OSF.IO/G8ZTP.

## Supplementary Materials

The following supporting information can be downloaded at https://www.mdpi.com/article/10.3390/brainsci16050480/s1. File S1: Search Strategy. PRISMA_2020_abstract_checklist. PRISMA_2020_checklist.
