# Supplementary material for: Efficacy of Non-Invasive Brain Stimulation in Improving Working Memory in Children and Adolescents with Attention-Deficit/Hyperactivity Disorder: A Systematic Review
Source: Brain Sci. 2026 Apr 29;16(5):480. doi: 10.3390/brainsci16050480 (PMC13204134; doi:10.3390/brainsci16050480)
Supplement: Supplementary file 1 [file brainsci-16-00480-s001.zip › Search Strategy.pdf]

Search Strategy

SCOPUS

TITLE-ABS-KEY ((ADHD* OR attention deficit hyperactivity disorder) AND (transcranial direct current stimulation* OR repetitive transcranial magnetic stimulation*) AND (working memory*)).

Total Results: 94

Web of Science

TS= ((ADHD* OR attention deficit hyperactivity disorder) AND (transcranial direct current stimulation* OR repetitive transcranial magnetic stimulation*) AND (working memory*)).

Total Results: 99
